# Lower energy expenditures in infants from obese biological mothers

**DOI:** 10.1186/1475-2891-7-15

**Published:** 2008-05-16

**Authors:** Russell Rising, Fima Lifshitz

**Affiliations:** 1 EMTAC Inc., Santa Barbara, California, USA

## Abstract

**Background:**

Previous studies in adults have found that a lower resting metabolic rate is a predictor of future body weight gain.

**Methods:**

To determine if energy expenditures are reduced in infants born to obese mothers, 21 healthy infants (3.9 ± 1.9 months) born to lean (n = 7, BMI < 25 kg/m^2^), overweight (n = 7, BMI between 25–30) and obese (n = 7, BMI>30) mothers, respectively, participated in this study. Measurements of infant weight, length and skin-fold thicknesses, and mother's weight and height were obtained. Infant energy expenditure was measured for 4-hours using the Enhanced Metabolic Testing Activity Chamber. Metabolic data were extrapolated to 24-hours and adjusted for differences in age and body composition using linear regression analysis (SPSS, version 13) and expressed as kcal/day. Differences between the three groups were determined by one way ANOVA with the Bonferroni Post Hoc test procedure (p < 0.05).

**Results:**

Infants born to obese mothers had a greater BMI (16.7 ± 1.2) than those from both the overweight (15.3 ± 1.4, p < 0.05) and lean groups (15.1 ± 1.3; p < 0.05). The infants of obese mothers had greater body fat (26.8 ± 2.1) than those from the overweight group (22.4 ± 5.0, p < 0.06). Infant BMI correlated (r = 0.53; p < 0.01) with that of their mothers. Extrapolated 24-h EE (kcal/d) correlated with fat-free mass (r = 0.94; p < 0.01). Infants extrapolated 24-h EE from both obese (472.1 ± 30.7 kcal/d; p < 0.05) and overweight groups (471.8 ± 39.5; p < 0.05) were lower than those of the lean group (532.4 ± 30.7).

**Conclusion:**

Lower extrapolated 24-h energy expenditure was present in infants of overweight and obese biological mothers during the first three to six months of life. Furthermore, these infants showed increased BMI and body fat. If these changes are unchecked future childhood obesity may result.

## Background

Body composition of biological mothers might influence that of their offspring. For example, maternal obesity was associated with infant body fat [1] and greater subcutaneous adipose tissue in infants [2]. Furthermore, a greater maternal BMI during the first trimester of pregnancy was related to a higher prevalence of obesity in children two to four years of age [3]. It was also reported that a greater maternal BMI was a modest predictor of their daughter's relative weight at five years of age [4].

There are several possible factors of infant and childhood obesity not necessarily related to maternal obesity per se. For example, toddlers of obese mothers are more food compliant thus increasing energy intake [5]. Furthermore, there was an association of both maternal overweight and obesity with breast feeding duration [6]. We have reported that infants of obese mothers consume more energy in a shorter period of time and are fed less frequently compared to those born to normal weight mothers [7]. However, large maternal weight loss resulting from obesity surgery prevented the transmission of obesity to children [8].

Indirect whole body calorimeters have been utilized for metabolic studies in adults since the early 1980s [9]. It was reported that a lower metabolic rate predicts body weight gain in adults [10]. One of the first studies in infants to address metabolic changes in relation to future obesity was conducted by Roberts et al in 1988 [11]. These investigators reported that infants who became obese had lower total daily energy expenditure (EE) at three months of age due to a reduction in physical activity. Their study involved measurement of total daily energy expenditure by doubly-labeled water and postprandial metabolic rates by indirect calorimetry. Other studies utilizing similar techniques have reported inconsistent results. Wells et al [12] detected no relationship between total or sleeping energy expenditures and future indices of body fatness after 3.5 years of follow-up, but Stunkard et al [13] found both increased energy intake and total daily energy expenditures to be associated with increased body size at 2-years of age in infants born to obese mothers, though no relationship existed between infant sleeping metabolic rate and maternal obesity. The indirect calorimetry systems utilized in these studies [11-13] were not appropriate for infant studies due to the lack of a means for parental interaction and the short duration of one or two hours of metabolic measurements. Therefore, we conducted a well controlled metabolic study in infants from lean, overweight and obese biological mothers with a sophisticated indirect whole body calorimeter designed for clinical use in infants which allowed the measurements of all of the components of energy expenditure during one four-hour metabolic testing session [7,14,15].

## Methods

### Subjects

Biological mothers of 21 healthy infants (3.9 ± 1.9 months) were recruited from two hospital outpatient wellness clinics for this study (mean +/- SD anthropometrics of the group were: body weight 6.6 ± 1.4 kg and length 64.4 ± 6.8 cm). They were grouped according to their biological mothers BMI (kg/m^2^), being < 25 (lean), between 25–30 (overweight) and >30 (obese), respectively. The maternal BMI was utilized for this classification due to its high positive correlation with body fat [16,17]. This resulted in seven infants each from lean, overweight and obese mothers, respectively (Table 1). None of the infants studied was born prematurely or had a low birth weight or intrauterine growth retardation. They were all of appropriate weight for gestational age and born full term. None of the infants were below the 5^th ^percentile for weight and length according to the revised NCHS growth charts [18]. All infants were of Hispanic ethnic origin except two in the overweight group whose parents were Caucasian and one each in the overweight and obese groups, respectively, whose parents were Afro-American.

**Table 1:** Anthropometrics of infants grouped according to maternal BMI

	7, (4M/3F) Lean	7, (3M/4F) Overweight	7, (4M/3F) Obese
Maternal BMI (kg/m^2^)	21.8 ± 1.9	27.4 ± 1.2 *	38.0 ± 6.2‡†
Age (months)	4.0 ± 1.5	3.5 ± 2.4	4.2 ± 2.1
Body weight (kg)	6.7 ± 1.3	6.1 ± 1.4	7.0 ± 1.5
Length (cm)	66.3 ± 5.7	62.9 ± 7.3	63.9 ± 8.0
Body fat (%)	23.8 ± 4.1	22.4 ± 5.0	26.8 ± 2.1 ‡
BMI (kg/m^2^)	15.1 ± 1.3	15.3 ± 1.4	16.7 ± 1.2 †‡
Weight for length (percentile)	17.1 ± 18.0	28.6 ± 32.5	53.6 ± 37.4†
Weight for age (percentile)	55.7 ± 31.1	55.7 ± 33.8	71.4 ± 15.5
Length for age (percentile)	87.6 ± 10.4	73.6 ± 34.8	67.1 ± 36.7
Standard deviation score for body weight	0.19 ± 0.90	-0.22 ± 1.00	0.44 ± 1.02

* = (p < 0.05) between the lean and over weight groups

† = (p < 0.05) between the lean and obese groups

‡ = (p < 0.05) between the over weight and obese groups

Prior to the study the health status of the infant was verified by a standard physical exam [19]. Infants were excluded if they had fever, diarrhea or any illness in the preceding 24-hours prior to the study. Parents were provided with a complete explanation regarding the purpose, procedure, risks and benefits of the study and informed consent was obtained from at least one parent of each infant. The study was approved by both the Institutional Review Boards of Miami Children's Hospital and Sansum Diabetes Research Institute/Cottage Hospital of Santa Barbara.

### Anthropometry

Prior to the metabolic study supine length (crown to heel, cm) was measured in duplicate with a horizontal stadiometer (Perspective Enterprises, Kalamazoo, MI) and body weight (kg) was the average of three measurements obtained with an infant scale (Cardinal Detecto, Webb City, MO). Body fat (%) and fat-free mass (kg) was calculated from body weight and length measurements according to formulas developed for infants [lnFFM = log transformed fat-free mass = 0.433 + (0.056 × (Square root (body weight, kg × length, cm)] [20]. Percent fat was the difference between the log transformed fat-free mass and body weight. Growth performance in regards to weight-for-length, weight-for-age and length-for-age percentiles were obtained from sex appropriate NCHS growth charts [18]. Standard Deviation Score (SDS) for weight was also calculated according to the formula [SDS = ((body weight, kg - 6.421)/1.43)]. All anthropometric and growth data are shown in Table 1.

### Brief description of the Enhanced Metabolic Testing Activity Chamber (EMTAC)

**Figure 1:**
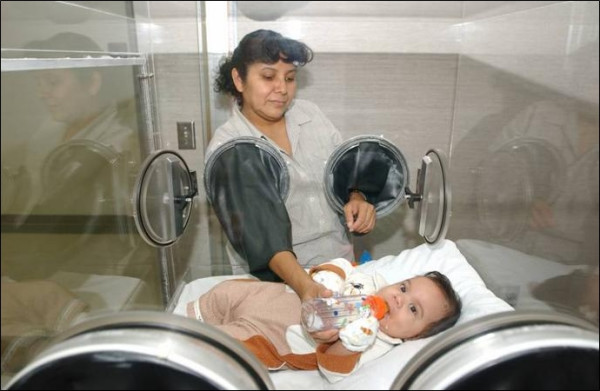
Photo of "natural" maternal-to-infant interaction during metabolic measurements.

A more detailed description of the EMTAC, validation for use in infants and how the components of energy expenditure are determined were described in previous studies [14,15,21]. One of the unique features incorporated into the EMTAC was the hand access ports that were installed around the entire enclosure. This allowed access to the infant under close to natural conditions (skin-skin contact) for normal care without corrupting the environment within the enclosure (Figure 1). Another unique feature was the installation into the infant enclosure of a Mettler balance (Model PMK-30, Mettler-Toledo AG, Greifensee, Switzerland). This balance was put under the platform that the infant was placed on and it provided a continuous digital output, which was used for accurate detection and recording of physical activity. The computer software corrected the volume of the EMTAC enclosure to account for the presence of the person's arms [21].

### Energy expenditure

Mothers were given instruction on how to interact with their infants and allowed time to practice using the hand access ports prior to metabolic testing. Once all of the instruction and calibrations were complete each infant was placed in the EMTAC, beginning at 9:00 AM, for four-hours of continuous measurements of energy expenditure (kcal/min), physical activity (oscillations in weight/min/kg body weight) and the respiratory quotient (RQ:VCO_2_/VO_2_). The oscillations in weight used to determine physical activity was derived from the balance that was placed under the infant during the metabolic test. The test was begun before infant feeding in accordance to their usual schedule. Any supplies such as diapers, formula, baby food or toys were placed in the EMTAC in hanging bags before the start of the test. Parents continued to feed their infants at their discretion during the four-hour metabolic test. Eight of the infants were fed Carnation^® ^Good Start, five with Enfamil^® ^with iron and eight with Similac Advance^® ^with iron. Caloric intake by the infants during the four-hour metabolic test was determined by the amount of formula consumed utilizing calibrated bottles and the formula manufacturer proximate analysis for energy. Investigators who were involved with the study acted as observers and recorded all infant activities such as feedings, periods of observed sleep and amount of parental interaction during the entire metabolic testing period as described previously [14,15,21].

Energy expenditure was continuously calculated during metabolic testing according to the method of Jequier [22] and summarized every five minutes as described previously [15,21,23]. At the conclusion of each metabolic test, all metabolic data were corrected for parental interaction [21]. The percentage of time infants were observed to be asleep was calculated by dividing total minutes of observed sleep by 240 (minutes in 4-hours). The result was multiplied by 100. Sleeping metabolic rate (SMR; kcal/day) was than computed by taking the mean energy expended (kcal/min) across all periods of observed infant sleep and multiplying the result by 1440 (minutes in 24 hours). The calculations for SMR are similar to the methodology used for adults [9] and validated for infants as reported in previous studies [14,21].

### Statistical analysis

The sample size was determined in advance [24], based on data obtained by utilizing short-term metabolic measurements in the EMTAC in previous studies [14,15,21,23]. The number of subjects studied was sufficient to detect differences, with a probability of 5%, in 24-hour extrapolated energy expenditure between the three groups of infants. With a standard deviation of 44.7 kcal/day, a difference greater than 33 kcal/day in 24-hour extrapolated energy expenditure or sleeping metabolic rates between any of the three groups was considered significant [24].

After extrapolation to 24-hours, metabolic data were adjusted for differences in age and body composition by regression analysis utilizing the anthropometric data of the 21 infants in this study. Extrapolated 24-hour energy expenditure (kcal/day) and sleeping metabolic rate (kcal/day) were the dependant variables in each of their own equations while fat-free mass (kg), percent body fat (%) and age (months) were the independent variables. Both equations and related statistical parameters are shown in Table 2. Thereafter, the residuals for 24-h extrapolated energy expenditure and sleeping metabolic rates were calculated by taking the differences between the raw and calculated values. Finally, adjusted values were calculated by adding the residual for each metabolic value to the respective group mean [9,10].

**Table 2:** Derived equations that were utilized to adjust infant metabolic data for body size and composition

Equations	R^2^	SE	P
24-h Energy expenditure = [-340.2 + (157.9 × Kg FFM*) + (23.4 × AGE* in months) + (-1.7 × % body fat)]	0.90	62.8	<0.001
Sleeping metabolic rate = [-192.2 + (74.7 × Kg FFM*) + (20.1 × AGE* in months) + (6.1 × % body fat*)]	0.93	34.1	<0.001

* = Coefficient a significant predictor (p < 0.05)

All anthropometric and adjusted metabolic data were analyzed utilizing one-way ANOVA (SPSS, version 13). Due to the number of infants in each group, differences between the lean, overweight and obese groups were determined by the Bonferroni Post Hoc test procedure (p < 0.05). All data were expressed as mean ± standard deviation unless otherwise noted.

## Results

### Anthropometrics

**Figure 2:**
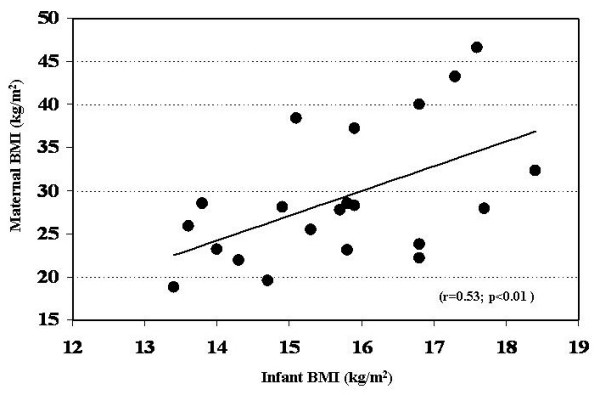
Relationship between infant and maternal BMI.

There were no significant differences in regards to age, length and body weight among the three groups of infants when classified according to their biological mothers BMI (Table 1). However the BMI of infants of obese mothers was greater (p < 0.05) as compared with both their overweight and lean counterparts. The body fat percentage in the infants of obese mothers was also greater in comparison to those in the overweight (p < 0.06) and lean groups (Table 1). The weight-for-length percentile differences were more than double (p < 0.05) between the lean and obese infants. Moreover, there was a positive correlation between the maternal BMI (r = 0.53; p < 0.01) and that of their infants (Figure 2). Even though not significant, there was a trend for a progressive increase in the standard deviation score for body weight between those infants in the lean (0.19) and those in the obese (0.43) groups (Table 1).

### Energy Metabolism

**Figure 3:**
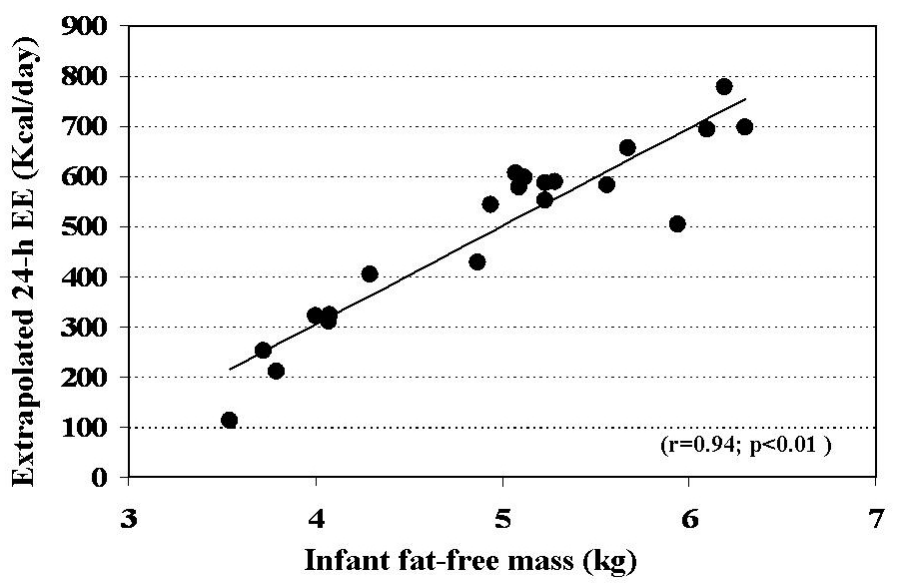
Relationship between unadjusted extrapolated 24-h energy expenditure and fat-free mass.

There was a strong positive correlation (r = 0.94; p < 0.01) between fat-free mass and 24-hour extrapolated energy expenditure (Figure 3). A similar positive relationships with fat-free mass was found for sleeping metabolic rate (r = 0.93; p < 0.01). Fat-free mass and age were significant coefficients (p < 0.05) in the regression equation utilized to adjust 24-h energy expenditure while all three coefficients (fat-free mass, fat mass and age) were significant predictors in the regression equation for adjustment of sleeping metabolic rate (Table 2).

The extrapolated 24-hour EE (p < 0.05) was lower in both groups of infants from overweight and obese mothers in comparison to their lean counterparts (Table 3). There were no differences in sleeping metabolic rate adjusted for fat-free mass, fat mass and age among the three groups of infants (Table 3). Moreover, nutrient utilization was not different in regards to increasing maternal BMI (Table 3). There were also no significant differences in energy intake among the three groups of infants during the course of the four-hour metabolic measurement, though infants of obese mothers appeared to consume more energy. Moreover, infants of obese mothers slept less (p < 0.05) than those from the overweight group (Table 3). Finally, no differences were found in physical activity among the three groups of infants (Table 3).

**Table 3:** Energy metabolism, physical activity and the amount of time asleep during the metabolic test in infants grouped according to maternal BMI

	Lean	Overweight	Obese
Number of infants	7	7	7

Energy intake during 4-hour study (kcal)	127.2 ± 91.4	136.6 ± 71.2	220.4 ± 127.2
24-hour energy expenditure (kcal/day)^1^	532.4 ± 30.7	471.8 ± 39.5*	472.1 ± 76†
Sleeping metabolic rate (kcal/day)^1^	407.5 ± 24.7	407.5 ± 37.2	402.9 ± 35.8
Sleeping time during metabolic test (%)	39.3 ± 21.6	50.0 ± 20.9	21.4 ± 11.1‡
Respiratory quotient (VCO_2_/VO_2_)	0.86 ± 0.04	0.84 ± 0.05	0.85 ± 0.08
Physical activity (Oscillations/min/kg BW)	4.3 ± 1.7	6.8 ± 7.8	7.6 ± 2.4

^1 ^Extrapolated to 24-hours and corrected for differences in body composition and age

* = (p < 0.05) between the lean and over weight groups

† = (p < 0.05) between the lean and obese groups

‡ = (p < 0.05) between the over weight and obese groups

## Discussion

In this study we demonstrated that infants born to overweight and obese biological mothers exhibited lower extrapolated 24-h EE than those of lean mothers. In other studies parental obesity was associated with a lower resting metabolic rate in children [25], though others did not find any decline in the resting metabolic rate in already obese adolescents [26]. We also found that 3–6 month old infants born to obese mothers had a greater percentage of body fat along with greater weight-for-length and weight-for-age percentiles. Moreover, the BMI of the infants was related to that of their mothers. We utilized maternal BMI for classifying the infants since it is a well established criterion for determining obesity in adults [16] and is highly correlated with body fat in both children and adults [27].

The Infants in the obese group spent the least amount of time asleep during the metabolic test. This might be due to them spending more time feeding as evidenced by the greater amount of energy consumed. Furthermore, the metabolic test was conducted between the hours of 9:00 AM and 1:00 PM where sleeping and feeding habits might differ in comparison to that during evening or night periods. Moreover, the lack of differences in the respiratory quotient and the amount of physical activity among the three groups of infants suggest that other metabolic or physiologic components might account for the reduction of extrapolated 24-h energy expenditure. In a previous 24-hour metabolic study, infants born to obese mothers slept more, consumed a greater portion of their energy intake as carbohydrate and tended to have a greater respiratory quotient than their lean counterparts [14]. It is possible that over a 24-hour period, greater sleeping time and more carbohydrates being oxidized to lipids might be contributing to a lower overall metabolic rate. Moreover, it is possible the greater carbohydrate intake might reduce the thermic effect of food thus also contributing to a reduced metabolic rate [28]. However, it is very difficult to measure this parameter accurately in infants. It is possible that the shorter duration of this study might have masked these contributions to a lower metabolic rate.

It is possible that the lower extrapolated 24-h EE found in infants of obese mothers might be an underlying contributing factor to the relationships between childhood and adult BMI's found in previous studies [3,4,27]. Moreover, two additional reports described that if children were in the upper percentiles (85–95^th^) for BMI, there was a probability of greater than 80% that they would be obese at age 35 [27,29]. Finally, the association between infantile and maternal BMI was found in a large cohort study where over 52,306 infants were followed up after birth [30]. The recent studies of Kral et al [8] regarding the prevalence of obesity of children from mothers who underwent weight reduction through bariatric surgery suggest an abrogation of the expression of genetic factors in offsprings in post severely obese mothers and that external factors may be more significant in determining weight gain of their children.

In our previous study [7] where maternal obesity and infant feeding interactions were recorded during 24-hour metabolic measurements in the EMTAC, infants born to obese mothers were shown to consume more energy in a shorter period of time than their leaner counterparts. Obese biological mothers also spent less time interacting with their babies than their normal weight counterparts. The 24-hour measurements of food intake are evidently more accurate and reliable indicator of total energy intake than the four-hour measurement performed in this experiment, though the infants of obese mothers appeared to ingest more energy and were spending more time awake even in a brief period of observation.

We have found in a previous study, utilizing infants from similar ethnic backgrounds, that at least four-hours are necessary to obtain an accurate measurement of infant metabolic rate [14]. Due to employment obligations and other commitments, the parents of the infants in our study were unable to stay for a longer period of measurement, hence we utilized the minimum time of four-hours that provided valid results [14]. To date we have studied over 150 infants with various clinical conditions in the EMTAC [14,15,21,23,31]. All of the mothers reported feeling comfortable with utilizing the hand access ports when caring and feeding their infants. This probably reduced, or eliminated, the separation anxiety and/or discomfort of the infants that was associated with previous measurements of metabolic rate [11,32,33].

In regards to energy metabolism, extrapolated 24-h EE and sleeping metabolic rates were both highly positively correlated with fat-free mass. This has been shown in many previous studies in adults [9,10] and infants [14,15,21]. This is suggestive that fat-free mass is the main metabolic component of the body. However, none of these relationships had an intercept equal to zero; therefore, this necessitated the adjustment of the energy expenditure data for differences in body composition and age utilizing regression analysis, a mathematical methodology that has been validated in adults [9,10] and in infants [21]. Moreover, body weight alone cannot be used to adjust 24-h EE because the relationship between the two parameters does not pass through zero. This is due to different proportions of metabolically active muscle mass in relation to the assumed non-metabolically inactive fat-mass in same sized individuals, thus causing errors in the adjustment of 24-h EE [9,10].

Actual feeding practices and the relationship to obesity have been studied for decades. In a recent review of 3600 publications in the area of infant feeding and related cardiovascular risk factors [34], it was found that breast feeding, especially for a longer duration, was found to be protective against future obesity. The infant feeding method and obesity in the Avon longitudinal study of parents and children showed that there was a protective association with attenuation of fat-free mass when breast feeding was prolonged for over six months [35] while maternal feeding restriction was an important factor determining the effects of breast feeding on future overweight [36]. Other studies [37,38] found that obese mothers of a low social class were more likely to formula feed thus leading to obesity in their offspring. In our study, all infants were formula fed, and the resulting increase in body fatness seems to coincide with the results of previous studies [33,37,38].

Most of our study population was from Hispanic origin. This is in contrast to the mainly Caucasian subjects in some of the previous studies of the relationship between metabolic rate and obesity [25-27]. It has been reported in another previous study that resting metabolic rate is lower in Afro-American boys when compared to their Caucasian counterparts. Moreover, Afro-American girls had lower total daily and activity energy expenditures than Caucasians of similar age and sex [39]. It is possible that infants from certain ethnic backgrounds begin to show metabolic characteristics that might predispose them to future childhood obesity right from the time of birth.

The onset of obesity can occur in any infant, regardless of ethnic background, that has a lower than average metabolic rate. Knowing which factors play a role in determining which infant is prone to excess body weight gain may allow early lifestyle interventions in order to prevent the future onset of childhood obesity. Future studies with a larger number of infants from Caucasian, and other ethnic groups, need to be conducted in order to determine which infants show metabolic changes that might predispose them to future childhood obesity. Finally, our results indicate that lifestyle interventions should begin right from the time of birth in those individuals predisposed to future obesity.

## Conclusion

We utilized a single comprehensive method to assess daily energy expenditure in 3–6 month old infants and found that infants born to overweight and obese mothers had lower 24-hour energy expenditure, increased BMI and increased body fat when compared to infants born to normal weight mothers.

## Abbreviations

EMTAC: Enhanced metabolic testing activity chamber; ANOVA: Analysis of Variance; BMI: Body mass index; 24-h EE: Twenty-four hour extrapolated energy expenditure; RMR: Resting metabolic rate; SMR: Sleeping metabolic rate; PA: Physical activity index; RQ: Respiratory quotient; NCHS: National Centers for Health Statistics; SDS: Standard Deviation Score; BW: Body weight; M: Males; F: Females.

## Competing interests

The authors declare that they have no competing interests.

## Authors' contributions

RR has contributed to the design of the experiment and conducted the data analysis. Furthermore, he either participated in some of the actual data acquisition or supervised pediatric research fellows in this regard. He also assisted in the preparation of the small grants necessary for funding of this project. Finally, he also assisted in the writing and editing of this manuscript.

FL directed the research and contributed to the preparation of the manuscript and assisted with data analysis. He also generated some of the grant proposals necessary for the financial support of this study. Both authors were involved in the final writing of this manuscript.

## References

[B1] Sewell MF, Houston PL, Super DM, Catalano P (2006). Increased neonatal fat mass, not lean body mass, is associated with maternal obesity. Am J Obstet Gynecol.

[B2] Forsum E, Lof M, Olausson H, Olhager E (2006). Maternal body composition in relation to infant birth weight and subcutaneous adipose tissue. Br J Nutr.

[B3] Whitaker RC (2004). Predicting preschooler obesity at birth: the role of maternal obesity in early pregnancy. Pediatrics.

[B4] Birch LL, Fisher JO (2000). Mothers' child-feeding practices influence daughters' eating and weight. Am J Clin Nutr.

[B5] Lumeng JC, Burk LM (2006). Maternal prompts to eat, child compliance, and mother and child weight status. J Pediatr.

[B6] Oddy WH, Li J, Landsborough L, Kendall GE, Henderson S, Downie J (2006). The association of maternal overweight and obesity with breastfeeding duration. J Pediatr.

[B7] Rising R, Lifshitz F (2005). Relationship between maternal obesity and infant feeding-interactions. Nutr J.

[B8] Kral JG, Biron S, Simard S, Hould FS, Lebel S, Marceau S, Marceau P (2006). Large maternal weight loss from obesity surgery prevents transmission of obesity to children who were followed for 2–18 years. Pediatrics.

[B9] Ravussin E, Lillioja S, Anderson TE, Christin L, Bogardus C (1986). Determinants of 24-hour Energy expenditure in man: Methods and results using a respiratory chamber. J Clin Invest.

[B10] Ravussin E, Lillioja S, Knowler WC, Christin L, Freymond D, Abbott WG, Boyce V, Howard BV, Bogardus C (1988). Reduced rate of energy expenditure as a risk factor for body weight gain. N Engl J Med.

[B11] Roberts SB, Savage J, Coward WA, Chew B, Lucas A (1988). Energy expenditure and intake in infants born to lean and overweight mothers. N Engl J Med.

[B12] Wells JC, Stanley M, Laidlaw AS, Day JM, Davies PS (1996). The relationship between components of infant energy expenditure and childhood body fatness. Int J Obes Relat Metab Disord.

[B13] Stunkard AJ, Berkowitz RI, Schoeller D, Maislin G, Stallings VA (2004). Predictors of body size in the first 2 y of life: a high-risk study of human obesity. Int J Obes Relat Metab Disord.

[B14] Rising R, Duro D, Cedillo M, Valois S, Lifshitz F (2003). Daily metabolic rate in healthy infants. J Peds.

[B15] Duro D, Rising R, Cedillo M, Lifshitz F (2002). Association between colic history in the first three months of life and carbohydrate malabsorption from fruit juices in infancy. Ped.

[B16] Calle EE, Thun MJ, Petrelli JM, Rodriguez C, Heath CW (1999). Body-mass index and mortality in prospective cohort of U.S. adults. N Eng J Med.

[B17] Roche AF, Sievogel RM, Chumlea WC, Webb P (1981). Grading of body fatness from limited anthropometric data. Am J Clin Nutr.

[B18] CDC Growth Charts (2000). National Center for Health Statistics. Health, United States, 2000 Hyattsville.

[B19] Guidelines for Health Supervision III (1997). American Academy of Pediatrics.

[B20] De Bruin NC, Van Velthoven KA, Stijnen T, Juttmann RE, Degenhart HJ, Visser HK (1995). Body fat and fat-free mass in infants: new and classic anthropometric indexes and prediction equations compared with total-body electrical conductivity. Am J Clin Nutr.

[B21] Cole C, Rising R, Mehta R, Hakim A, Choudhury S, Sundaresh M, Lifshitz F (1999). Comprehensive assessment of the components of energy expenditure in infants using a new infant respiratory chamber. J Am Coll Nutr.

[B22] Jequier E, Bjoerntorp P, Cairella M, Howard A (1981). Long-term measurement of energy expenditure in man: direct and indirect calorimetry. Recent advances in obesity research Libbey, London.

[B23] Cole C, Rising R, Lifshitz F (1999). Are there consequences of incomplete carbohydrate absorption from fruit juice consumption in infants?. Arch Pediatr Adolesc Med.

[B24] Kuzma JW (1998). Basic statistics for the health sciences.

[B25] Griffiths M, Payne PR, Stunkard AJ, Rivers JP, Cox M (1990). Metabolic rate and physical development in children at risk of obesity. Lancet.

[B26] Bandini LG, Schoeller DA, Dietz WH (1990). Energy expenditure in obese and nonobese adolescents. Pediatr Res.

[B27] Guo SS, Wu W, Chumlea WC, Roche AF (2002). Predicting overweight and obesity in adulthood from body mass index values in childhood and adolescence. Am J Clin Nutr.

[B28] Larson DE, Rising R, Ferraro RT, Ravussin E (1995). Spontaneous overfeeding with a "cafeteria diet" in men: Effects on 24-hour energy expenditure and substrate oxidation. Int J Obes.

[B29] Najjar MF, Rowland M (1987). Anthropometric reference data and prevalence of overweight, United States, 1976–80. Vital Health Stat II.

[B30] Stettler N, Zemel BS, Kumanyika S, Stallings VA (2002). Infant weight gain and childhood overweight status in a multicenter, cohort study. Pediatrics.

[B31] Valois S, Rising R, Duro D, Cole C, Cedillo M, Lifshitz F (2003). Carbohydrate malabsorption may increase daily energy requirements in infants. Nutrition.

[B32] Moon JK, Jensen CL, Butte NF (1993). Fast-response whole body indirect calorimeters for infants. J Appl Physiol.

[B33] Butte NF, Moon JK, Wong WW, Hopkinson JM, Smith EO (1995). Energy requirements from infancy to adulthood. Am J Clin Nutr.

[B34] Owen CG, Martin RM, Whincup PH, Smith GD, Cook DG (2005). Effect of infant feeding on the risk of obesity across the life course: a quantitative review of published evidence. Pediatrics.

[B35] Toschke AM, Martin RM, von Kries R, Wells J, Smith GD, Ness AR (2007). Infant feeding method and obesity: body mass index and dual-energy X-ray absorptiometry measurements at 9–10 y of age from the Avon Longitudinal Study of Parents and Children (ALSPAC). Am J Clin Nutr.

[B36] Taveras EM, Rifas-Shiman SL, Scanlon KS, Grummer-Strawn LM, Sherry B, Gillman MW (2006). To what extent is the preventive effect of breast feeding on future overweight explained by decreased maternal feeding restriction?. Pediatrics.

[B37] Hediger ML, Overpeck MD, Kuczmarski RJ, Ruan WJ (2001). Association between infant breastfeeding and overweight in young children. JAMA.

[B38] Li L, Parson TJ, Power C (2003). Breastfeeding and obesity in childhood: cross sectional study. BMJ.

[B39] DeLany JP, Bray GA, Harsha DW, Volaufova J (2002). Energy expenditure in preadolescent African American and white boys and girls: the Baton Rouge Children's Study. Am J Clin Nutr.

